# Supplementary figures and images for: Microbiota in the ptarmigan intestine—An Inuit delicacy and its potential in popular cuisine
Source: PLoS One. 2024 Dec 23;19(12):e0305317. doi: 10.1371/journal.pone.0305317 (PMC11666028; doi:10.1371/journal.pone.0305317)

Supplementary Fig. S1


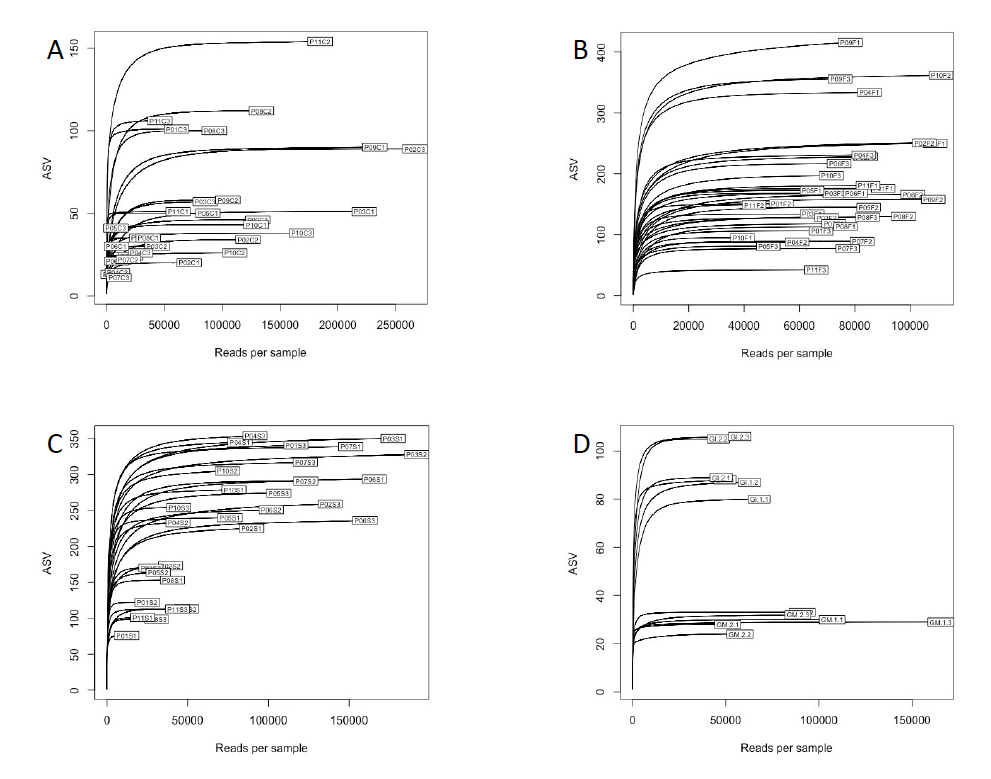

Supplement: S1 Fig — Rarefaction curve of the sample types of crop (A), stomach (B), intestines (C) and the two types of garum meat and intestines (D). The curves confirm a sufficient sequencing depth to cover the microbial diversity found in the samples as they reach asymptote. (DOCX) [file pone.0305317.s001.docx]
