# Supplementary material for: Microbiota in the ptarmigan intestine—An Inuit delicacy and its potential in popular cuisine
Source: PLoS One. 2024 Dec 23;19(12):e0305317. doi: 10.1371/journal.pone.0305317 (PMC11666028; doi:10.1371/journal.pone.0305317)
Supplement: S1 Table — (DOCX) [file pone.0305317.s002.docx]

**Supplementary Table S1.** Tasting attributes for the two garum types used for CATA.

| Salty | Unbalanced flavor | Black olives |
| --- | --- | --- |
| Sweet | Mouth-watering | Game flavor |
| Sour | Flavor | Liver flavor |
| Umami | New | Yeasty |
| Bitter | Traditional | Caramelized |
| Fish sauce | Luxury | Cheesy |
| Soy sauce | Satisfying | Dry mouth-feel |
| Grain flavor | Healthy |  |
| Intense flavor | Artificial |  |
| Mild flavor | Alcoholic odor |  |
| Rich flavor | Fermented flavor |  |
| Balanced flavor | Synthetic flavor |  |
| Astringent | Muddiness |  |
