# Supplementary material for: Microbiota in the ptarmigan intestine—An Inuit delicacy and its potential in popular cuisine
Source: PLoS One. 2024 Dec 23;19(12):e0305317. doi: 10.1371/journal.pone.0305317 (PMC11666028; doi:10.1371/journal.pone.0305317)
Supplement: S2 Table — P-value of the alpha diversity metrics richness, faith phylogenetic diversity, Shannon diversity index and Simpsons diversity index. Pairwise Wilcoxon test was performed. P value of 0.05 is set with the p-value adjusted for False Discovery Rate method. (DOCX) [file pone.0305317.s003.docx]

**Supplementary Table S2.** The p-value for the pairwise Wilcoxon test of the alpha diversity indices.

Richness

| Pairwise Wilcoxon test | Crop | Stomach | Intestines | Garum Intestines |
| --- | --- | --- | --- | --- |
| Stomach | 5.6 × 10^-9^* |  |  |  |
| Intestines | 1.16 × 10^-8^* | 0.02015* |  |  |
| Garum Intestines | 0.01003* | 0.00279* | 0.02057* |  |
| Garum Meat | 0.62536 | 0.00038* | 0.00038* | 0.00833* |

* Significant

Faith phylogenetic diversity

| Pairwise Wilcoxon test | Crop | Stomach | Intestines | Garum Intestines |
| --- | --- | --- | --- | --- |
| Stomach | 5.9 × 10^-9^* |  |  |  |
| Intestines | 1.85 × 10^-8^* | 0.01527* |  |  |
| Garum Intestines | 0.01004* | 0.00042* | 0.01527* |  |
| Garum Meat | 0.56659 | 3.08 x 10^-6^* | 2.04 × 10^-6^* | 0.00361* |

* Significant

Shannon diversity index

| Pairwise Wilcoxon test | Crop | Stomach | Intestines | Garum Intestines |
| --- | --- | --- | --- | --- |
| Stomach | 8.2 × 10^-9^* |  |  |  |
| Intestines | 0.00049* | 0.00049* |  |  |
| Garum Intestines | 0.0801 | 0.00056* | 0.62655 |  |
| Garum Meat | 0.20959 | 0.00049* | 0.23652 | 0.06854 |

* Significant

Simpson diversity index

| Pairwise Wilcoxon test | Crop | Stomach | Intestines | Garum Intestines |
| --- | --- | --- | --- | --- |
| Stomach | 2.5 × 10^-7^* |  |  |  |
| Intestines | 0.0031* | 0.0014* |  |  |
| Garum Intestines | 0.1459 | 0.0033* | 0.8455 |  |
| Garum Meat | 0.1936 | 0.0012* | 0.6357 | 0.1077 |

* Significant
