# Supplementary material for: Microbiota in the ptarmigan intestine—An Inuit delicacy and its potential in popular cuisine
Source: PLoS One. 2024 Dec 23;19(12):e0305317. doi: 10.1371/journal.pone.0305317 (PMC11666028; doi:10.1371/journal.pone.0305317)
Supplement: S3 Table — PERMANOVA results for the bacterial community clustering based on Unifrac unweighted distance. P value of 0.05 is set with the p-value adjusted with Bonferroni for multiple comparisons. (DOCX) [file pone.0305317.s004.docx]

**Supplementary Table S3.** PERMANOVA results of the dissimilarity between the bacterial community clustering based on the Unifrac unweighted distance.

| Comparison | Df | SumsOfSqs | F.Model | R2 | p.value | p.adjusted |
| --- | --- | --- | --- | --- | --- | --- |
| Crop vs. Intestines | 1 | 2.9657 | 11.6876 | 0.1607 | 0.001 | 0.01* |
| Crop vs Stomach | 1 | 3.5310 | 14.3559 | 0.2011 | 0.001 | 0.01* |
| Crop vs. Garum Intestines | 1 | 1.2075 | 4.27470 | 0.1116 | 0.001 | 0.01* |
| Crop vs. Garum Meat | 1 | 1.5710 | 5.79498 | 0.1456 | 0.001 | 0.01* |
| Intestines vs. Stomach | 1 | 0.3844 | 1.96094 | 0.0316 | 0.16 | 0.16 |
| Intestines vs. Garum Intestines | 1 | 0.9962 | 5.01661 | 0.1193 | 0.001 | 0.01* |
| Intestines vs. Garum Meat | 1 | 2.3069 | 12.2628 | 0.2489 | 0.001 | 0.01* |
| Stomach vs. Garum Intestines | 1 | 1.0855 | 6.08347 | 0.1556 | 0.001 | 0.01* |
| Stomach vs. Garum Meat | 1 | 2.4937 | 14.9574 | 0.3118 | 0.001 | 0.01* |
| Garum Intestines vs. Garum Meat | 1 | 0.9881 | 14.9574 | 0.4763 | 0.005 | 0.05* |

* Significant
