# Supplementary material for: Microbiota in the ptarmigan intestine—An Inuit delicacy and its potential in popular cuisine
Source: PLoS One. 2024 Dec 23;19(12):e0305317. doi: 10.1371/journal.pone.0305317 (PMC11666028; doi:10.1371/journal.pone.0305317)
Supplement: S4 Table — P-value of the tasting attributes. The appropriate statistical comparison of 2 groups either Cochran Q test or Fishers exact test was performed. P-value of 0.05 is set. (DOCX) [file pone.0305317.s005.docx]

**Supplementary Table S4.** CATA results of the two garum types.

| Flavor | Sum intestines | | Sum Meat | | Test | p-value |
| --- | --- | --- | --- | --- | --- | --- |
|  | Yes | No | Yes | No |  |  |
| Salty | 5 | 7 | 9 | 3 | Cochran Q | 0.097 |
| Sweet | 10 | 2 | 5 | 7 | Fisher’s | 0.089 |
| Sour | 3 | 9 | 0 | 12 | Fisher’s | 0.2173 |
| Umami | 12 | 0 | 10 | 2 | Fisher’s | 0.4783 |
| Bitter | 0 | 12 | 0 | 12 | Fisher’s | 1 |
| Fish sauce | 5 | 7 | 3 | 9 | Fisher’s | 0.668 |
| Soy sauce | 6 | 6 | 8 | 4 | Cochran Q | 0.4076 |
| Grain flavor | 10 | 2 | 5 | 7 | Fisher’s | 0.0893 |
| Intense flavor | 6 | 6 | 6 | 6 | Cochran Q | 1 |
| Mild flavor | 0 | 12 | 3 | 9 | Fisher’s | 0.2174 |
| Rich flavor | 11 | 1 | 8 | 4 | Fisher’s | 0.3168 |
| Balanced flavor | 2 | 10 | 2 | 10 | Fisher’s | 1 |
| Astringent | 2 | 10 | 3 | 9 | Fisher’s | 1 |
| Unbalanced | 0 | 12 | 0 | 12 | Fisher’s | 1 |
| Mouth-watering | 6 | 6 | 9 | 3 | Fisher’s | 0.4003 |
| Familiar | 0 | 12 | 2 | 10 | Fisher’s | 0.4782 |
| New | 3 | 9 | 0 | 12 | Fisher’s | 0.2174 |
| Traditional | 7 | 5 | 2 | 10 | Fisher’s | 0.0893 |
| Luxury | 0 | 12 | 2 | 10 | Fisher’s | 0.4783 |
| Satisfying | 5 | 7 | 3 | 9 | Fisher’s | 0.6668 |
| Healthy | 0 | 12 | 0 | 12 | Fisher’s | 1 |
| Artificial | 0 | 12 | 0 | 12 | Fisher’s | 1 |
| Alcohol odor | 0 | 12 | 0 | 12 | Fisher’s | 1 |
| Fermented flavor | 9 | 3 | 12 | 0 | Fisher’s | 0.2174 |
| Synthetic flavor | 0 | 12 | 0 | 12 | Fisher’s | 1 |
| Muddiness | 0 | 12 | 0 | 12 | Fisher’s | 1 |
| Black olives | 0 | 12 | 0 | 12 | Cochran Q | 3.4E-7* |
| Game flavor | 10 | 2 | 0 | 12 | Cochran Q | 0.0041* |
| Liver flavor | 0 | 12 | 0 | 12 | Fisher’s | 1 |
| Yeasty | 3 | 9 | 0 | 12 | Fisher’s | 0.2174 |
| Caramelized | 10 | 2 | 0 | 12 | Fisher’s | 0.4873 |
| Cheesy | 8 | 4 | 8 | 4 | Fisher’s | 1 |
| Dry mouthfeel | 0 | 12 | 0 | 12 | Fisher’s | 1 |

* Significant
