# Supplementary material for: Microbiota in the ptarmigan intestine—An Inuit delicacy and its potential in popular cuisine
Source: PLoS One. 2024 Dec 23;19(12):e0305317. doi: 10.1371/journal.pone.0305317 (PMC11666028; doi:10.1371/journal.pone.0305317)
Supplement: Attachment — Submitted filename: Response to Reviewers.docx [file pone.0305317.s006.docx]

**Response to reviewers and editors of PLOS ONE**

We thank the reviewers and editors for their time, comments, and constructive suggestions. We provided responses to the reviewers’ comments below.

**General revisions:**

The formatting of the manuscript has been checked to meet PLOS ONE's formatting requirements.

The reference list was checked using scite.ai and showed no references with editorial

concern.

The data is currently being deposited at the NCBI SRA under ID SUB14679393

**Responses to reviewer 1 comments:**

Line 44: there is an open parenthesis but the close is missing – A closing parenthesis has been added at line 45.

Line 261: correct to 53.3% to make it consistent with other percentages – A punctuation (.) has replaced a comma (,) to make it consistent with the rest of the percentages.

Line 275: I'm not sure if Phylogenetic needs to be capitalized – Phylogenetic has been corrected to phylogenetic.

We have included the host-specificity of Helicobacter as recommended by the reviewer and thank the reviewer for relevant discussion point and references.

The following three references have been added to the reference list in connection to the above revision:

Dubois A, Berg DE, Incecik ET, Fiala N, Heman-Ackah LM, Del Valle J, et al. Host specificity of Helicobacter pylori strains and host responses in experimentally challenged nonhuman primates. Gastroenterology. 1999;116: 90–96. doi:10.1016/S0016-5085(99)70232-5

Ferrero RL, Jenks PJ. In Vivo Adaptation to the Host. In: Mobley HL, Mendz GL, Hazell SL, editors. Helicobacter pylori: Physiology and Genetics. Washington DC: ASM Press; 2001. pp. 583–592. doi:10.1128/9781555818005.ch46

Lee A, Fox J, Hazell S. Pathogenicity of Helicobacter pylori: a Perspective. Infect Immun. 1993; 1601–1610.

**Responses to reviewer 2 comments:**

Figure 2 has been given labels A, B, C, and D and the specific alpha diversity measures have been added at each Y-axis.
R2 has been added to the p-value as suggested.

In response to remark for lines 395-403 a brief section has been added, discussing the potential of the garum to enhance safety of consumption in combination with microbial richness.

We agree that it would be valuable to know more about the shared taxa between the intestines and the garum made with the intestines. We would like to explore this further in a follow-up study that allows for a broader statistical assessment and strain detection.
